# Heat-Inactivated *Pediococcus acidilactici* pA1c^®^HI Maintains Glycemic Control and Prevents Body Weight Gain in High-Fat-Diet-Fed Mice

**DOI:** 10.3390/ijms26136408

**Published:** 2025-07-03

**Authors:** Miriam Cabello-Olmo, María Oneca, Saioa Goñi, Raquel Urtasun, María José Pajares, Deyan Yavorov-Dayliev, Iñaki Iturria, Josune Ayo, Ignacio J. Encío, Miguel Barajas, Miriam Araña

**Affiliations:** 1 Biochemistry Area, Department of Health Science, Public University of Navarre, 31008 Pamplona, Spain; miriamcabelloolmo@gmail.com (M.C.-O.); saioa.goni@unavarra.es (S.G.); raquel.urtasun@unavarra.es (R.U.); mjose.pajares@unavarra.es (M.J.P.); ignacio.encio@unavarra.es (I.J.E.); 2 Genbioma Aplicaciones S.L., Office D3, Pl, CEIN, 31110 Noáin, Spaindeyan@genbioma.com (D.Y.-D.); iiturria@genbioma.com (I.I.); josune@genbioma.com (J.A.); 3 IDISNA Navarra′s Health Research Institute, 31008 Pamplona, Spain

**Keywords:** *Pediococcus acidilactici*, postbiotic, high-fat diet, diabetes, glycemia, obesity

## Abstract

Heat-inactivated probiotics, also known as postbiotics, have emerged as an alternative to live probiotics, and have been shown to be good therapeutic tools for the management and treatment of metabolic dysfunctions such as obesity and type 2 diabetes (T2D). The present study aimed to assess the anti-obesogenic and anti-diabetic properties of heat-inactivated *Pediococcus acidilactici* pA1c^®^ (pA1c^®^HI) in mice fed a high-fat diet (HFD). The animals were given an HFD or HFD enriched with either the probiotic alive pA1c^®^ or the postbiotic pA1c^®^HI. Body weight (BW), serum biochemical markers, gene expression, and histological changes were determined following 15 weeks of supplementation. The postbiotic pA1c^®^HI exerted a similar effect on glucose metabolism to that exerted by pA1c^®^ supplementation. Nevertheless, we observed that pA1c^®^HI supplementation maintained BW, attenuated adipogenesis, and protected the mice from liver damage more efficiently than pA1c^®^. Similarly, in adipose tissue, pA1c^®^HI significantly downregulated markers of de novo lipogenesis (DNL) and fat storage. The observed results show that pA1c^®^HI administration was even more effective in mitigating the HFD’s detrimental effects than pA1c^®^ supplementation, and therefore, the viability of this *Pediococcus acidilactici* CECT 9879 strain is not required for preserving its beneficial properties in the context of obesity and T2D.

## 1. Introduction

The intestinal microbiome is a collection of microbial genomes that inhabit the intestines and are closely related to host health due to their involvement in the host’s physiology, metabolism, and immune system [1,2]. Data from the scientific literature have proven that type 2 diabetes (T2D), a prevailing chronic metabolic disease, is associated with intestinal dysbiosis [3,4], and the same has also been reported for obesity [5,6]. This has led to great efforts to develop gut microbiome-targeted therapies to aid in the prevention or treatment of T2D and obesity.

To date, the most used biotherapeutics are probiotics, which are live microorganisms which confer a health benefit on the host when consumed in adequate amounts [7]. Numerous probiotic studies on T2D have already been reviewed and summarized elsewhere [8,9,10], highlighting their suitability as therapeutic tools for diabetes and obesity prevention and/or management [11,12]. Nevertheless, novel microbiota-targeted interventions, such as postbiotics and fecal material transplants, have recently begun to be developed and have been studied for T2D management, showing metabolic improvements as well [13,14,15]. Postbiotics are preparations of inanimate microbes that confer health benefits on hosts. They largely consist of intact microbial cells and/or structures; however, they can also include microbial biomass [16]. These compounds have been claimed to have beneficial effects [17] and, in many cases, induce effects comparable to those exerted by live probiotic cells, such as antimicrobial activity against potential pathogens, improvements in gut permeability, and immunomodulation [18,19,20]. Additionally, postbiotics are becoming increasingly popular among researchers and industry because they offer some advantages over probiotics, such as greater stability during storage, a reduced risk of the delivery of antibiotic-resistance genes, and a lower risk of developing adverse effects [20,21], making them a potential alternative to probiotics for high-risk population groups [22,23].

We have previously shown the anti-diabetic properties of the lactic acid bacteria *Pediococcus acidilactici* pA1c^®^ (pA1c^®^) on high-fat diet (HFD)-induced mice, which improves insulin resistance, maintains body weight, and also enhances the beneficial effects of metformin treatment [24,25]. However, the potential effects of heat-inactivated pA1c^®^ (pA1c^®^HI) have remained unknown. Considering the numerous benefits of postbiotics [17,20], the purpose of this study was to investigate pA1c^®^HI health benefits and compare its potential advantages over pA1c^®^ in HFD-fed mice.

## 2. Results

### 2.1. pA1c^®^HI Controlled Glucose Dysregulation in HFD-Fed Mice

**Figure 1:**
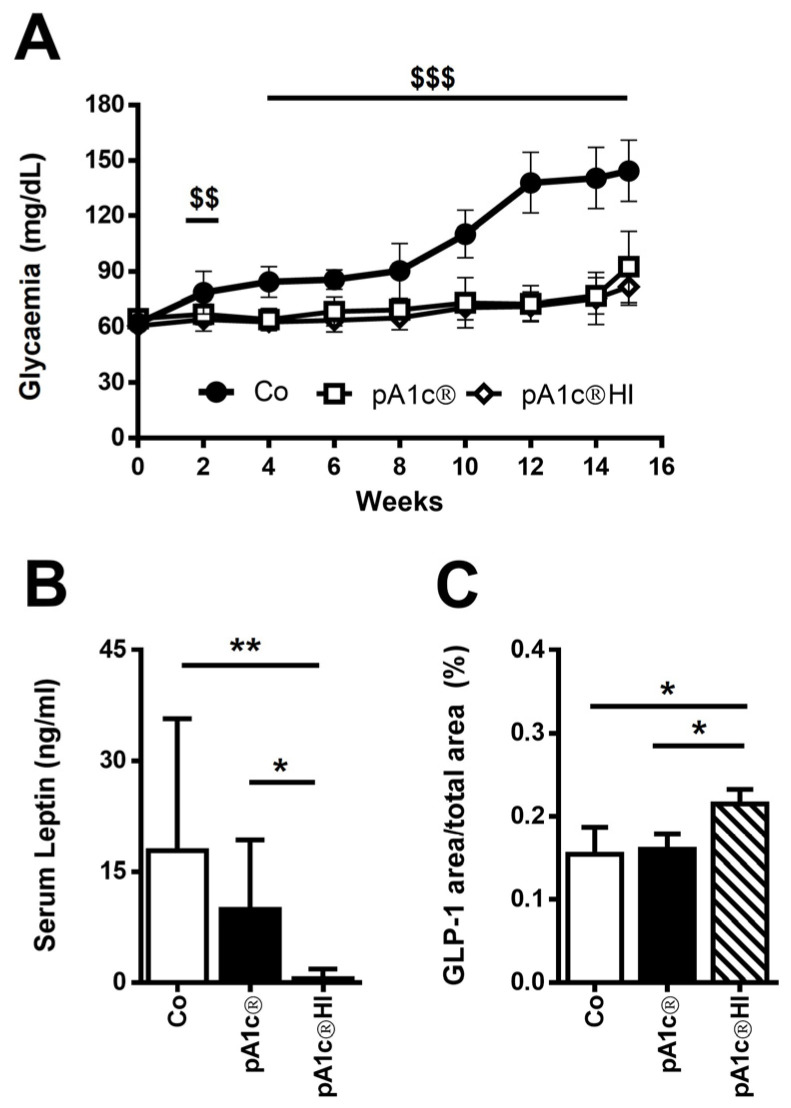
Effect of pA1c^®^ and pA1c^®^HI on (**A**) glycemia, (**B**) serum leptin, and (**C**) colonic GLP-1. *n* = 8–10 animals/group. ^$$^ *p* < 0.01 vs. Co; ^$$$^ *p* < 0.001 vs. Co; * *p* < 0.05; ** *p* < 0.01.

At the beginning of the study, fasting blood glucose (FBG) levels were similar between the groups (average FBG 62.4 ± 3.9 mg/dL, range 54.0–70.0 mg/dL). After starting the administration of the diets, FBG began to increase and continued to ascend in the control group (Co). However, in the mice in the pA1c^®^HI group, blood glucose levels remained constant for 15 weeks and were similar to those observed at the beginning of the study. We observed significant differences in glycemia levels after 2 weeks of supplementation with pA1c^®^HI with respect to the Co group (*p* < 0.05 in week 2 and *p* < 0.01 from week 4 to the end of the study) (Figure 1A).

Furthermore, the serum leptin levels of the pA1c^®^HI group were strongly inhibited when compared to the Co group (Co: 17.9 ± 17.8 ng/mL and pA1c^®^HI: 0.5 ± 1.3 ng/mL; *p* < 0.01) and pA1c^®^ group (pA1c^®^: 9.9 ± 9.4 ng/mL and pA1c^®^HI: 0.5 ± 1.3 ng/mL; *p* < 0.05). This indicates that postbiotic supplementation affects satiety. Thus, it is suggested that pA1c^®^HI intervention prevented the development of obesity and demonstrated that pA1c^®^HI is more effective than pA1c^®^ in the management of obesity (Figure 1B).

On the other hand, the immunohistochemical quantification of colonic GLP-1 revealed an increased GLP-1^+^ area in the pA1c^®^HI group compared to the other two groups (Co: 0.15 ± 0.03%, pA1c^®^: 0.16 ± 0.02%, and pA1c^®^HI: 0.22 ± 0.02%; *p* < 0.05) (Figure 1C).

### 2.2. pA1c^®^HI Prevented Body Weight Gain and Improved Obesity-Related Parameters in HFD-Fed Mice More Effectively than pA1c^®^

**Figure 2:**
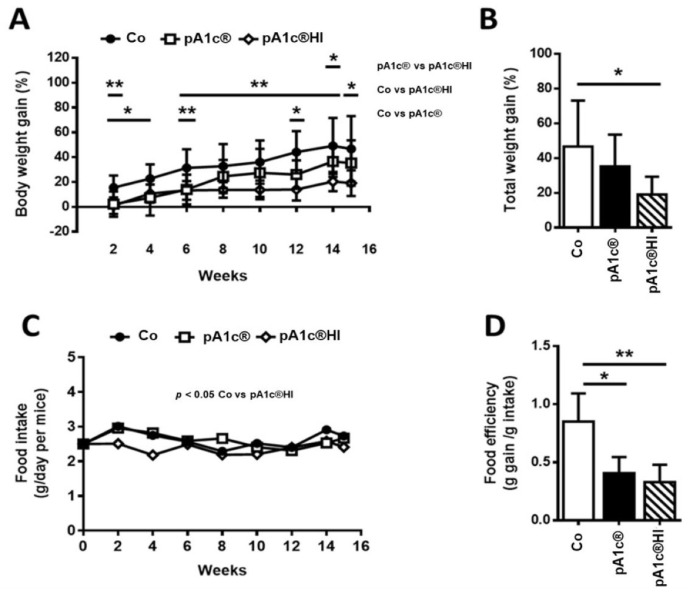
Effect of pA1c^®^ and pA1c^®^HI on (**A**) body weight gain (%), (**B**) total weight gain (%), (**C**) food intake, and (**D**) food efficiency. *n* = 9–10 animals/group. * *p* < 0.05 and ** *p* < 0.01.

After 15 weeks with the HFD, the body weight (BW) of the Co group increased by 46.7 ± 26.4%. However, supplementation with pA1c^®^HI led to a rise in BW of only 19.1 ± 10.3%. The animals supplemented with the postbiotic gained 40% less weight than the non-supplemented animals (Figure 2A). Moreover, Figure 2B shows that the total weight gained (%) at the end of the study was significantly lower (*p* < 0.05) with pA1c^®^HI vs. in the Co group, evidencing the strong anti-obesogenic effect of pA1c^®^HI.

Furthermore, statistically significant differences were also observed between the pA1c^®^HI and pA1c^®^ groups (*p* < 0.05) (Figure 2A), emphasizing once again that pA1c^®^HI is more effective in reducing total BW gain and modulating lipid metabolism than pA1c^®^.

Mice ate a significantly lower amount of feed when the diet was supplemented with pA1c^®^HI vs. the Co group (*p* < 0.05) (Figure 2C), suggesting that pA1c^®^HI could affect the satiety center of the animal via its activation. In addition, as shown in Figure 2D, we found differences in food efficiency (described as grams of weight gain/grams of food intake) between the control and supplemented animals (Co vs. pA1c^®^, *p* < 0.05; Co vs. pA1c^®^HI, *p* < 0.01). Therefore, this confirmed that BW control was related to postbiotic administration rather than variations in food intake.

**Figure 3:**
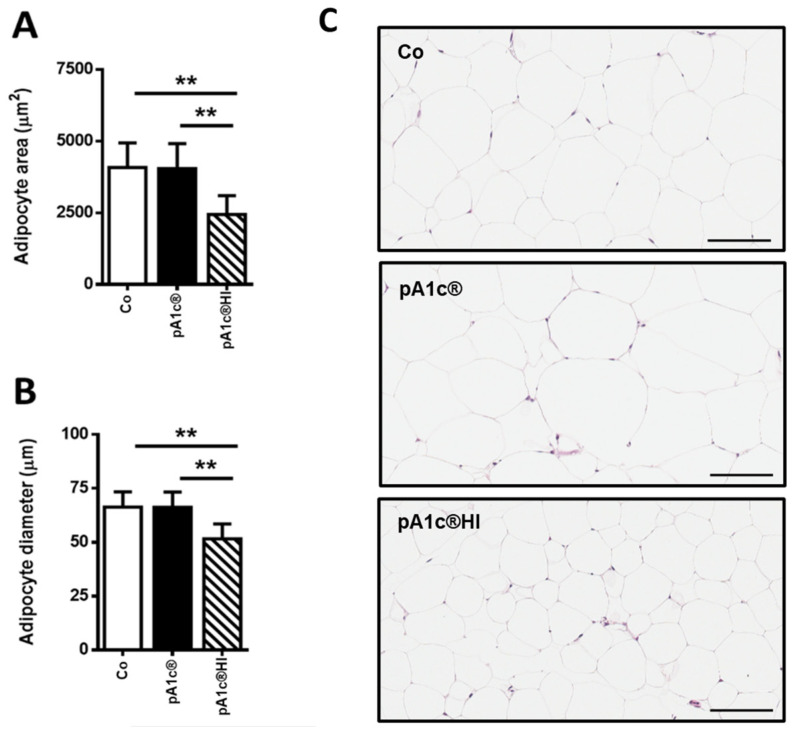
Effect of pA1c^®^ and pA1c^®^HI on (**A**) adipocyte area and (**B**) adipocyte diameter. (**C**) Representative images of H&E-stained adipose tissue sections (scale bars = 100 µm). *n* = 9–10 animals/group. ** *p* < 0.01.

We analyzed the area and diameter of adipocytes in mesenteric adipose tissue and found that the administration of pA1c^®^HI slowed the increase in adipocyte size (*p* < 0.01) (Figure 3A,B). This is directly related to the lower amount of adipose tissue in the pA1c^®^HI group (Figure 3C) and directly correlated with the lower BW gain, demonstrating once again the adipose tissue-reducing activity of pA1c^®^HI.

### 2.3. pA1c^®^HI Had a Protective Effect Against Liver Injury

**Figure 4:**
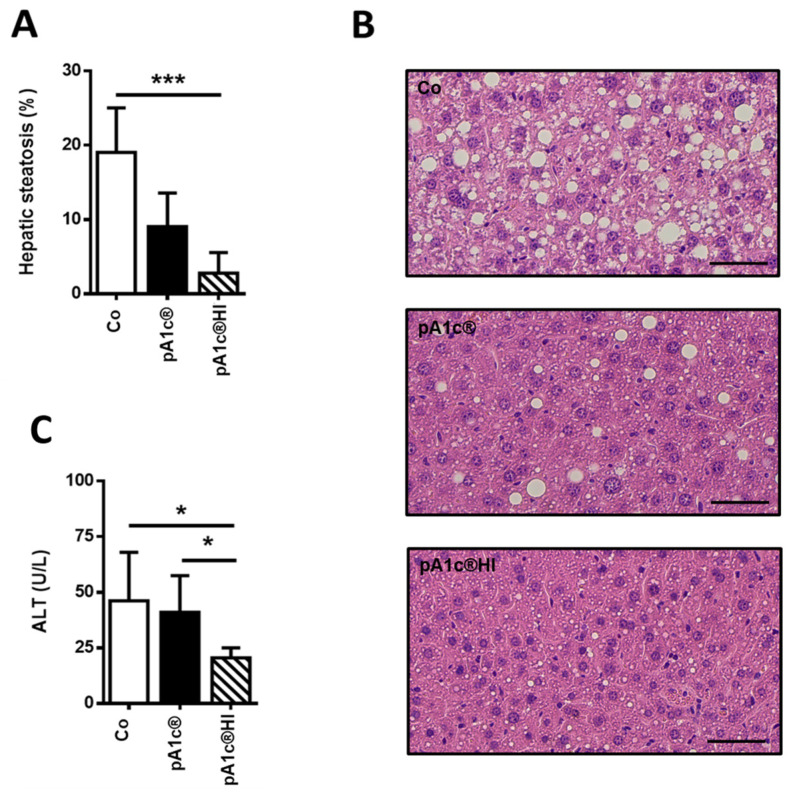
Impact of pA1c^®^ and pA1c^®^HI on (**A**) hepatic steatosis, (**B**) representative images of H&E-stained liver sections (scale bars = 50 µm), and (**C**) ALT serum levels. *n* = 9–10 animals/group. * *p* < 0.05 and *** *p* < 0.001.

The supplementation of pA1c^®^HI generated significantly lower levels of liver steatosis than in the Co group (Co: 19.0 ± 6.0% vs. pA1c^®^HI: 2.8 ± 2.8%; *p* < 0.05). Despite the fact that steatosis levels were lower in the pA1c^®^ group than in the Co group, significant differences were not found, probably due to data variability (Co: 19.0 ± 6.0% vs. pA1c^®^: 9.1 ± 4.5%; *p* > 0.05) (Figure 4A,B).

Moreover, we found a significant decrease in serum ALT levels in the pA1c^®^HI group vs. the Co group (*p* < 0.05), and the pA1c^®^HI group vs. the pA1c^®^ group (*p* < 0.05), with the lowest ALT levels in the pA1c^®^HI group (pA1c^®^HI: 20.5 ± 4.6 U/L, Co: 46.2 ± 21.8 U/L, and pA1c^®^: 41.1 ± 16.5 U/L) (Figure 4C).

### 2.4. Both pA1c^®^ and pA1c^®^HI Modulated Hepatic Lipid Metabolism, but Only pA1c^®^HI Had an Effect on Adipose Tissue Lipid Metabolism Gene Expression

**Figure 5:**
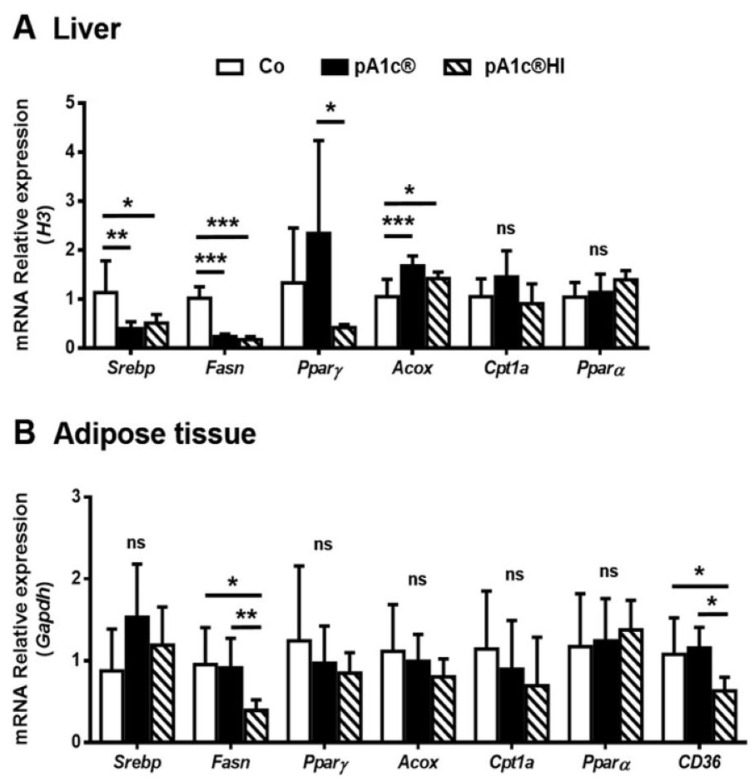
Impact of pA1c^®^ and pA1c^®^HI on (**A**) hepatic and (**B**) adipose tissue gene expression. *n* = 8 animals/group. * *p* < 0.05, ** *p* < 0.01, and *** *p* < 0.001. *Acox*: Acyl-coenzyme A oxidase 1; *Cpt1a*: Carnitine palmitoyl transferase deficiency—type 1; *CD36*: Cluster of differentiation 36; *Fasn*: fatty acid synthase; ns: no significant differences; *Pparα*: peroxisome proliferator-activated receptor α; *Pparγ*: peroxisome proliferator-activated receptor γ; *Srebp*: sterol regulatory element-binding protein.

In terms of liver analysis (Figure 5A), we can highlight that the expression of de novo lipogenesis (DNL) markers *Srebp* and *Fasn* was downregulated in both the pA1c^®^ (*p* < 0.01) and pA1c^®^HI groups (*p* < 0.05) when compared to the Co group. *Pparγ*, a lipid uptake and adipogenic marker, was notably upregulated with pA1c^®^ and significantly differed from the pA1c^®^HI group, which presented levels comparable to those in the Co group (*p* < 0.05). Such results, however, are the result of a substantial deviation in the data. Regarding beta-oxidation pathway markers, although we did not find significant differences for *Cpt1* or *Pparα* (*p* > 0.05), we found differences for *Acox*, whose expression was significantly higher in both the pA1c^®^ and pA1c^®^HI groups compared to the Co group (*p* < 0.001 and *p* < 0.05, respectively). In conclusion, both the probiotic and the postbiotic had similar metabolic effects in the liver, decreasing DNL and increasing the catabolic beta-oxidation pathway.

However, in the adipose tissue (Figure 5B), the DNL marker *Fasn* decreased only in the pA1c^®^HI group (pA1c^®^HI vs. Co, *p* < 0.05; and pA1c^®^HI vs. pA1c, *p* < 0.01), and no differences were found for *Srebp* or the lipid uptake marker *Pparγ* (*p* > 0.05). Unlike in the liver, no significant differences were found for the beta-oxidation pathway markers *Acox*, *Cpt1*, or *Pparα* (*p* > 0.05), but we did find significant differences for *CD36*, with its expression being lower in the pA1c^®^HI group compared to the Co and pA1c^®^ groups (*p* < 0.05). In summary, since *CD36* is involved in the import of fatty acids into cells, the administration of pA1c^®^HI decreased DNL and the store of fatty acids in adipose tissue cells.

### 2.5. pA1c^®^ and pA1c^®^HI Differentially Affected Gut Microbiota Composition

Because one outlier was detected in the Co group, the analysis of the microbiota was carried out with 14 samples instead of 15 (*n* = 4 in Co, and *n* = 5 in pA1c^®^ and pA1c^®^HI).

**Figure 6:**
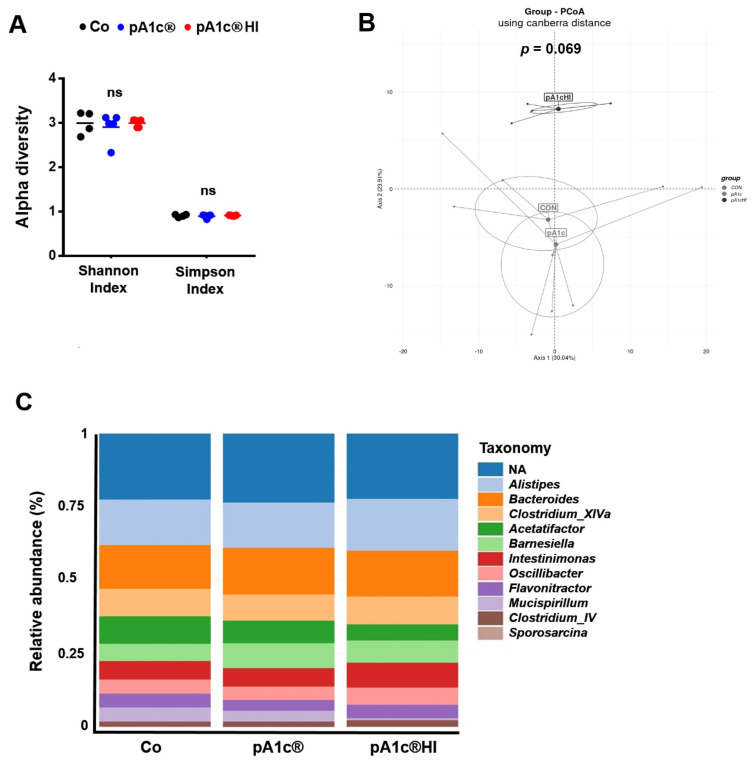
Treatment effects on mouse fecal microbiota. (**A**) Alpha diversity metrics, (**B**) β-diversity represented by Principal coordinate analysis (Canberra distance at genus level), and (**C**) taxonomic profiling at genus level. *n* = 4–5 animals/group. ns: no significant differences.

We analyzed the α-diversity of gut microbiota at the genus level, and the Shannon and Simpson indexes revealed no differences between groups (*p* > 0.05) (Figure 6A). In regard to the β-diversity, the statistical analysis using the PERMANOVA test showed no significant differences (*p* = 0.069), despite the fact that the Co and pA1c^®^ groups are grouped next to each other, while the pA1c^®^HI group is far from both (Figure 6B). In line with the diversity parameters, as can be seen in Figure 6C, the taxonomic profile analyzed at the genus level revealed no significant differences, with *Alistipes*, *Bacteroides*, and *Clostridium XIVa* being the most abundant genera.

**Figure 7:**
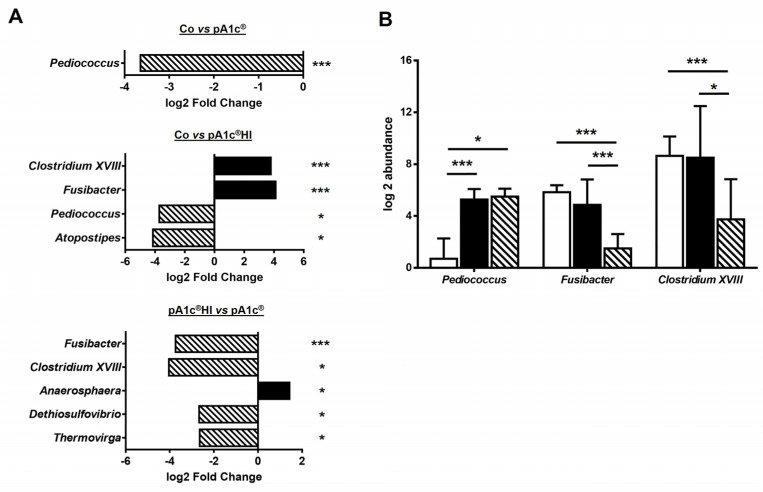
(**A**) Barplots showing fold-changes in comparisons between groups at the genus level. (**B**) Boxplots of relative abundance of bacterial taxa which significantly differ between groups. *n* = 4–5 animals/group. * *p* < 0.05 and *** *p* < 0.001.

Comparisons between groups were performed to further analyze the differences in the relative abundance of bacteria (Figure 7). Only *Pediococcus* taxon abundance significantly differed between the Co and pA1c^®^ groups (*p* < 0.001), and the same effect was found between the Co and pA1c^®^HI groups (*p* < 0.05). In addition, pA1c^®^HI consumption increased the abundance of another genus (*Atopostipes*) (*p* < 0.05) while decreasing that of two other genera (*Clostridium XVIII* and *Fusibacter*) (*p* < 0.001). Furthermore, five taxa were found to be significantly different between the pA1c^®^ and pA1c^®^HI groups. The postbiotic had a positive effect on *Fusibacter* (*p* < 0.001), *Clostridium XVIII* (*p* < 0.05), *Dethiosulfovibrio* (*p* < 0.05), and *Thermovirga* (*p* < 0.05) but a negative effect on *Anaerosphaera* (*p* < 0.05) in comparison with pA1c^®^.

## 3. Discussion

Owing to the rising number of subjects with overweight and obesity, a significant increase in T2D prevalence is expected in the coming years. Therefore, intensive research efforts have recently focused on developing non-pharmacological interventions as alternatives to classic treatments, including pro- and postbiotic formulations. In previous murine studies, it was shown that probiotic *P. acidilactici* pA1c^®^ (pA1c^®^) administration improves glucose tolerance [24,25,26] and prevents obesity-related alterations [27], which concurs with numerous publications on different *P. acidilactici* strains that reduce blood glucose levels and affect serum lipid levels [28,29,30]. Moreover, previous genomic analysis and characterization of *P. acidilactici* species provide strong evidence for its industrial development and clinical applications because of its safety profile and health benefits [30,31]. While collective data and publications (including meta-analysis) confirm the usefulness of probiotics in metabolic alterations [32,33], only scarce data on postbiotics are available [13,34]. In this context, and considering the inherent benefits of postbiotics compared to probiotics, we hypothesize that the postbiotic derived from heat-inactivated pA1c^®^ (pA1c^®^HI) could retain the beneficial effects observed for live pA1c^®^ and even offer some advantages.

Regarding the normoglycemic effect, which is one of the most important outcomes to consider in diabetes management, we observed that postbiotic supplementation had the same effect as the probiotic on FBG during the 15 weeks of the study, leading to markedly lower glycemic values in comparison with the untreated animals.

Previous systematic reviews and meta-analyses have suggested that probiotics’ effects on FBG could be, in part, mediated by their impact on GLP-1, a gut hormone that affects food digestion and insulin levels [33]. GLP-1 is known to have an important effect on glucose homeostasis [35], and several researchers have observed increased serum GLP-1 levels after probiotic administration in HFD-fed mice and other T2D preclinical models [24,36,37]. In this study, we have found that only supplementation with the postbiotic was effective in increasing GLP-1 intestinal levels.

Leptin is a peptide hormone synthesized mainly by white adipose tissue, and its concentration in plasma is typically proportional to fat mass [38,39]. Accordingly, we observed differences in the leptin levels between the pA1c^®^HI group and the Co and pA1c^®^ groups. Obesity is associated with high levels of leptin, and whether hyperleptinemia induces leptin resistance or not is controversial [40,41]. Zhao et al. [42] showed that through a reduction in the amount of leptin secreted by adipose tissue, mice are protected from HFD-induced obesity. This suggests that protection against hyperleptinemia may be important in preventing diet-induced obesity. This seems to contradict the model that leptin decreases food intake and increases energy expenditure. Indeed, we observed lower food intake and lower leptin levels due to the administration of the postbiotic, which was consistent with the finding that this group had the lowest BW and also the highest levels of colonic GLP-1, which is known to be involved in satiety and thus affects food intake [43,44]. Moreover, it is demonstrated that low leptin levels are associated with a greater capacity for weight loss in subjects with obesity [45]. In contrast, other studies have shown that leptin reduction increases the metabolic consequences of high-fat diet exposure [46,47]. Taken together, this data indicates that postbiotic supplementation affects satiety and food intake and prevents the development of T2D-related disturbances such as obesity, demonstrating that pA1c^®^HI is more effective than pA1c^®^ in the management of obesity.

With respect to the BW gain, the supplementation of pA1c^®^ resulted in reduced weight gain, although this effect was not significant after week 12, aligning with one previous study [24] but not with another [25]. Conversely, the use of pA1c^®^HI led to a sustained significant reduction in weight gain until the end of the study. Furthermore, when we analyzed the differences between groups at the end of the study, we observed a lower body weight gain in the pA1c^®^HI group compared to the control, emphasizing that pA1c^®^HI is more effective in reducing total BW gain and modulating lipid metabolism than pA1c^®^. This seemed to be related to the lower amount of adipose tissue, since, studying the size and area of the adipocytes, we observed smaller adipocytes in the pA1c^®^HI group, and it is known that an increase in the number and size of adipocytes causes adipose tissue expansion, which in turn can lead to obesity [48].

Regarding the hepatic lipid metabolism pathways, we observed that the supplementation of both the probiotic and the postbiotic decreased DNL through downregulating *Srebp* and *Fasn* expression and activated peroxisomal beta-oxidation by increasing *Acox* expression. Additionally, *P. acidilactici* FZU106 administration in hyperlipidemic rats significantly regulated the mRNA levels of liver genes involved in lipid metabolism and reduced steatosis [49]. In addition, treatments with different postbiotics were also related to nonalcoholic fatty liver disease (NAFLD) prevention and adipose tissue accumulation [50,51,52]. When we analyzed the accumulation of fatty acids in the liver, we also observed a lower degree of steatosis in the pA1c^®^HI-supplemented animals. On the other hand, regarding the regulation of lipid metabolism in adipose tissue, no effect was observed in animals when pA1c^®^ was administered, while animals treated with pA1c^®^HI had lower DNL, as we observed in the liver. In a model of mice fed a high-fat and high-sucrose (HFS) diet, the administration of pA1c^®^ for 10 weeks induced an increase in *Acox* expression [26], and in a model of rats with obesity induced by the same diet, fatty acid beta-oxidation was activated in the adipose tissue and the liver [27]. Furthermore, we found a lower expression of *CD36* when pA1c^®^HI was administered, which implies the reduced entry of fatty acids into the cell, favoring reduced storage in the adipose tissue, as observed in the size and area of the adipocytes in these animals. In conclusion, the results demonstrate that pA1c^®^HI is more effective than pA1c^®^ in modulating lipid metabolism.

Surprisingly, pA1c^®^HI consumption increased the abundance of the genus *Atopostipes* while decreasing *Clostridium XVIII* and *Fusibacter* in comparison with Co animals. A literature review showed that the genus *Fusibacter* is abundant in the intestinal microbiota of animals with greater body weight [53]. It is possible that pA1c^®^HI prevented an HFD-associated increase in *Fusibacter* in the animals supplemented with the postbiotic. Additionally, *Clostridium XVIII* is known to be more abundant in individuals with obesity compared to controls. Notably, treatments that reduce the abundance of *Clostridium XVIII* have been positively correlated with improvements in BW and body mass index in patients with obesity [54,55]. Consistent with this, our study found that the group with the lowest abundance of *Clostridium XVIII* (pA1c^®^HI) also exhibited the best body composition regulation and weight loss.

Overall, the results support the hypothesis that the postbiotic exerts beneficial effects on metabolism through multiple complementary mechanisms: the stimulation of GLP-1 secretion, modulation of the gut microbiota, improvement in intestinal barrier function, inhibition of lipogenesis, activation of oxidative pathways, and favorable immunomodulation. One of the most relevant findings is the increased colonic expression of GLP-1, an incretin hormone secreted by intestinal L-cells. This effect could be attributed to the direct action of postbiotic metabolites (such as short-chain fatty acids or bacterial structural components) on specific receptors of L-cells, promoting their activation and hormone secretion. Given the well-known effects of GLP-1 on satiety, insulin sensitivity, and inflammation reduction, this mechanism could help explain several of the observed effects, including the reduced food intake, BW loss, and improvements in hepatic and adipose tissue metabolism. Furthermore, a reduction in serum leptin levels, smaller mesenteric adipocyte area and size, and lower *CD36* expression in adipose tissue were observed. These changes suggest a decrease in fatty acid uptake and storage, along with reduced DNL and the potential reprogramming of adipose tissue toward a more functional phenotype. At the hepatic level, the observed profile (reduced lipogenesis, increased fatty acid oxidation, and decreased steatosis) is associated with improved insulin sensitivity and reduced lipotoxic stress. From an immunometabolic perspective, the postbiotic appears to have contributed to reduced systemic and intestinal inflammation. The decrease in pro-inflammatory bacterial genera such as *Clostridium* and *Fusibacter* suggests a more favorable gut environment, with lower endotoxemia and reduced activation of inflammatory pathways. The observed reduction in peripheral tissue inflammation, together with elevated GLP-1 levels and fat mass loss, fosters an environment that enhances insulin signaling. These effects may underlie the metabolic improvements associated with postbiotic administration, reinforcing its therapeutic potential for managing chronic metabolic disorders such as obesity and T2D.

According to the literature, there are two previous studies on murine [56] and human cell lines [57] and just one double-blind, randomized, controlled clinical trial [58] using heat-inactivated *P. acidilactici*. To the best of our knowledge, our study and a recently published study [59] in which we used the same strain are the first two in vivo studies addressing the topic of glucose or body weight control with the referenced postbiotic.

These results open the door to future clinical studies where the effects of pA1c^®^HI supplementation will help us confirm the beneficial effect of pA1c^®^HI in individuals at risk of developing obesity, diabetes, or other metabolic dysfunctions.

## 4. Materials and Methods

### 4.1. Experimental Design

A total of 30 male C57BL/6 mice (Charles River Laboratories), aged 9 weeks, underwent a two-week acclimation period on a standard chow diet. The animals were randomly divided and allocated into the following groups (*n* = 10 each): (1) the control group (Co), with animals receiving the HFD; (2) the pA1c^®^ group, with animals receiving the HFD plus a probiotic formulation with pA1c^®^, and (3) the pA1c^®^HI group, with animals receiving the HFD plus a postbiotic formulation with heat-inactivated (HI) pA1c^®^ (pA1c^®^HI).

During the study, all groups had ad libitum access to the diets and water. At the end of the study (15 weeks), all animals were sacrificed by cervical dislocation for the collection of tissue samples (liver, mesenteric adipose tissue, and colon) and blood. Tissue samples for quantitative real-time PCR were cut and frozen at −80 °C. Tissue samples for histological analysis were washed with PBS and fixed in a formaldehyde solution (10%) for 24 h. Blood samples were centrifuged for 8 min at 2000 rpm, and serum samples were stored at −80 °C until analysis.

All procedures involving animals were approved by the Institutional Committee on Care and Use of Laboratory Animals (CEEA, University of Navarra) (protocol number: CEEA/017-20).

### 4.2. Diets and Postbiotic Preparation

The probiotics/postbiotics were mixed with the HFD every 2 weeks under sterile conditions and kept at 4 °C until use. The HFD (TD.06414, Envigo, Tekla, Sparks, NV, USA) contained 60% of kcal from fats (detailed information in Supplementary Table S1). The dietary dose of pA1c^®^ was adjusted to 1 × 10^10^ CFU per animal per day, and the microbial load was verified using the standard plate count method (incubation at 37 °C with 5% CO_2_ for 48 h). The dose of pA1c^®^HI in the diet was adjusted to 1 × 10^10^ cells per animal per day.

The probiotic strain pA1c^®^, previously evaluated in several studies [24,25,26,60], is the subject of a patent application titled “Probiotics for regulating blood glucose [PCT/EP2020/087284; WO2021/123355A1]” and is deposited in the Spanish Type Culture Collection (CECT) under reference number CECT9879. This strain is considered safe under the Qualified Presumption of Safety (QPS) framework established by the European Food Safety Authority (EFSA). The postbiotic pA1c^®^HI was obtained by the heat inactivation method. After 30 min at 80 °C with constant stirring, non-viable cells were obtained with a maximum of 1 × 10^3^ CFU/g viable cells.

### 4.3. Body Weight and Fasting Blood Glucose

Body weight and 12 h FBG were determined every 2 weeks. FBG was measured using a glucometer (Accu-chek Aviva, Roche, Basel, Switzerland), and blood samples were obtained from the tip of the tail vein.

### 4.4. Serum Biochemical Analysis

At the end of the study, the serum ALT transaminase level was determined using the Cobas c-311 analyzer (Roche Diagnostics, Basel, Switzerland), and serum leptin concentration was measured by commercial ELISA kits (Abyntek Biopharma, Derio, Spain).

### 4.5. Histological Analysis

Formalin-fixed tissue samples (liver, colon, and adipose tissue) were embedded in paraffin, sectioned at a 3 µm thickness, and stained with hematoxylin and eosin (H&E) for light microscopy examination. To detect GLP-1 in colon tissue sections, an anti-GLP-1 antibody (ab26278, Abcam, Cambridge, UK) was used at a dilution of 1:2000. Detailed information on the protocol used for tissue sample preparation and immunolabeling is described in [24].

Liver and colon slides were digitized using the histology slide scanner Aperio CS, operating under Scan Scope Console software (v.10.2.0.2352, Leica Biosystems, Nussloch, Germany), and further analyzed using Fiji software (Fiji v.2.3.0 with ImageJ 1.53q). In H&E-stained liver sections, we analyzed steatosis (expressed as a percentage of the area occupied by lipid droplets) in ten random images (20×) per animal. In colon sections, we measured the percentage of GLP-1-positive cells per total cells in ten random fields (20×) per animal.

In H&E-stained adipose tissue sections, we evaluated adipocyte area and diameter with Adiposoft software (v.1.16, CIMA, University of Navarra, Pamplona, Spain).

### 4.6. Quantitative Real-Time PCR (RT-qPCR)

Frozen liver and adipose tissues stored at −80 °C were used for total RNA extraction using the RNeasy Mini Kit (Qiagen, Barcelona, Spain) according to the manufacturer’s protocol. RNA purity and concentration were assessed by spectrophotometry with a Nanodrop One (Thermo Scientific, Thermo Fischer Scientific, Madrid, Spain). Complementary DNA (cDNA) synthesis was performed using SuperScript^TM^ IV VILO^TM^ RT Premix with enzDNase^TM^ (Invitrogen, Thermo Fisher Scientific, Madrid, Spain) following the manufacturer’s protocol. Subsequently, mRNA expression levels of genes related to lipid metabolism were analyzed. The results were further normalized using histone H3 (*H3*) or glyceraldehyde-3-phosphate dehydrogenase (*Gapdh*) as a house-keeping gene for liver or adipose tissue, respectively. Quantitative real-time PCR (qPCR) was performed using the IQ SYBR Green Supermix (Bio-Rad Laboratories, Madrid, Spain) on a CFX96 Real-Time System (Bio-Rad Laboratories, Madrid, Spain). Data are expressed as relative mRNA levels normalized to *H3* (liver) or *Gapdh* (adipose tissue) and were analyzed using the comparative cycle threshold method (2^−∆∆CT^). Primer sequences for the targeted mouse genes and their sources are listed in Supplementary Table S2.

### 4.7. Fecal Microbiota Analysis

Stool samples were collected (*n* = 5 per group) after the 15-week period and frozen at −80 °C for microbiota analysis as previously described [25].

### 4.8. Statistical Analysis

Data are presented as the mean ± standard deviation (SD). All statistical procedures were performed using GraphPad Prism 8.0.1 software. Data normality was assessed using the Shapiro–Wilk and Kolmogorov–Smirnov tests. For datasets that met the assumption of normality, comparisons between groups were performed using one-way analysis of variance (ANOVA) followed by Tukey’s post hoc test. For data that did not follow a normal distribution, the non-parametric Kruskal–Wallis test was applied, followed by Dunn’s post hoc test for multiple comparisons.

All data groups were analyzed using parametric tests, except for serum leptin, steatosis, liver *Srebp*, adipose tissue *Fasn*, and adipose tissue *Pparα* determinations, which were analyzed using the non-parametric tests previously described.

Additionally, for the microbiota analysis, α-diversity was assessed using the Shannon and Simpson indexes. β-diversity was evaluated through Permutational Multivariate Analysis of Variance (PERMANOVA) based on Bray–Curtis dissimilarities to test for differences in microbial community structure among groups. Relative abundances of bacterial taxa were compared between groups to identify significant differences in genus-level composition. Statistical analyses were performed using SHAMAN as previously described [61].

Statistical significance was set at *p* < 0.05. * denotes *p* < 0.05, ** denotes *p* < 0.01, and *** denotes *p* < 0.001.

## 5. Patents

I.E., M.B., J.A., M.A., and M.O. are co-authors of the patent cited in the manuscript, “Probiotics for regulating blood glucose [PCT/EP2020/087284; WO2021/123355A1]”, and I.J.E., M.B., J.A., M.A., M.C.-O., D.Y.-D., I.I., and M.O. are co-authors of the patent “Postbiotic preparation for preventing or treating obesity [EP24382308.5; EP24219570.9; and 18/978,675 in the United States]”.

## Data Availability

The datasets generated or analyzed during the current study are available on request from the corresponding author.

## Supplementary Materials

The following supporting information can be downloaded at https://www.mdpi.com/article/10.3390/ijms26136408/s1 [62,63,64,65,66,67].
