# Supplementary material for: Heat-Inactivated Pediococcus acidilactici pA1c®HI Maintains Glycemic Control and Prevents Body Weight Gain in High-Fat-Diet-Fed Mice
Source: Int J Mol Sci. 2025 Jul 3;26(13):6408. doi: 10.3390/ijms26136408 (PMC12250037; doi:10.3390/ijms26136408)
Supplement: Supplementary file 1 [file ijms-26-06408-s001.zip › ijms-3710917-supplementary.pdf]

Table S1. Nutritional information TD.06414 (Envigo)

Formula	g/Kg	Composition	% by weight	% kcal from
Lard	310.0	Fat	34.3	60.3
Casein	265.0	Carbohydrate	27.3	21.4
Maltodextrin	160.0	Protein	23.5	18.3
Sucrose	90.0	Kcal/g	5.1	
Cellulose	65.5			
Mineral Mix, AIN-93G-MX (94046)	48.0			
Soybean Oil	30.0			
Vitamin Mix, AIN-93-VX (94047)	21.0			
L-Cystine	4.0			
Calcium Phosphate, dibasic	3.4			
Choline Bitartrate	3.0			
Blue Food Color	0.1			

Information obtained from Envigo Teklad Diets

Table S2. Sequences of primers used in gene expression analysis.

Target gene	Forward/ Reverse	Primer sequence (5´ to 3´)	Source
Acox	F	CTATGGGATCAGCCAGAAAG	[62]
	R	AGTCAAAGGCATCCACCAA	
CD36	F	CACAGCTGCCTTCTGAAATGTGTGG	[63]
	R	TTTCTACGTGGCCCGGTTCTAATTC	
Cpt1	F	TCTAGGCAATGCCGTTCAC	[63]
	R	GAGCACATGGGCACCATAC	
Fasn	F	AGCCATGGAGGAGGTGGTGAT	[64]
	R	GTGTGCCTGCTTGGGGTGGAC	
Gapdh	F	TCAAGAAGGTGGTGAAGCAG	[65]
	R	TCCACCACCCTGTTGCTGTA	
H3	F	AAAGCCGCTCGCAAGAGTGCG	[66]
	R	ACTTGCCTCCTGCAAAGCAC	
Pparα	F	ACAAGGCCTCAGGGTACCA	[62]
	R	GCCGAAAGAAGCCCTTACAG	
Pparγ	F	GCTGTTATGGGTGAAACTCTG	[67]
	R	GAATAATAAGGTGGAGATGCAGG	
Srepb	F	CACTTCATCAAGGCAGACTC	[64]
	R	CGGTAGCGCTTCTCAATGGC	

Acox: acetyl-coenzyme A oxidase 1; *CD36:* cluster of differentiation 36; *Cpt1:* carnitine palmitoyl-transferase 1; *Fasn*: fatty acid synthase; *Gapdh*: glyceraldehyde- 3-phosphate dehydrogenase; *H3*: Histone H3; *Pparα*: peroxisome proliferator-activated receptor *α; Pparγ*: peroxisome proliferator-activated receptor *γ; Srebp*: sterol regulatory element-binding protein.
